# Renal vein thrombosis due to metastatic germ cell tumor, report of a case with a very rare clinical scenario

**DOI:** 10.1002/cnr2.1910

**Published:** 2023-10-08

**Authors:** Hamidreza Ghorbani, Ali Emadi Torghabeh, Mahdi Farzadnia, Alireza Golshan, Parisa Rabiei

**Affiliations:** ^1^ Kidney Transplantation Complications Research Center Mashhad University of Medical Sciences Mashhad Iran; ^2^ Cancer Research Center Mashhad University of Medical Sciences Mashhad Iran; ^3^ Department of Pathology Mashhad University of Medical Sciences Mashhad Iran

**Keywords:** mixed germ cell tumor, renal vein thrombosis, teratoma, testicular neoplasms

## Abstract

**Background:**

Renal metastasis is a rare manifestation of germ cell tumors. Extension of malignant lesions into the renal vein can complicate the scenario.

**Case:**

This report presents a 35‐year‐old man with primary stage IS NSGCT. Fourteen months after radical orchiectomy he presented with metastasis in the lung, kidney, and para‐aortic lymph nodes. He received multiple lines of salvage treatments including chemotherapy and surgery. Intraoperative exploration during radical nephrectomy and retroperitoneal lymphadenectomy revealed intra‐renal vein involvement with a prominent teratomatous component.

**Conclusion:**

Defining the exact extent of malignant lesions, especially endovascular lesions, is very important to clarify how advanced the malignant lesions are. The surgeons must be aware of the risk factors that predict vascular involvement, and therefore, providing intraoperative access to vascular surgery procedures when needed.

## 1 INTRODUCTION

Testicular germ cell tumor (TGCT) accounts for only 1%–2% of male cancers, but it is the most common testicular cancer and the leading cause of cancer‐related death in adolescents and young men.^1^, ^2^ Invasive TGCT are thought to arise from testicular germ cells in situ (GCNIS) or intratubular germ cell tumors as a result of abnormal maturation of gonadal cells before or during the prenatal period.^3^ However, they are usually detected in the second and third decades of life.^1^ Mixed TGCTs account for a quarter of all testicular tumors and 30%–40% of all TGCTs and have various components, including embryonal carcinomas, yolk sac tumors, postpubertal teratomas, and choriocarcinoma.^4^, ^5^, ^6^ The retroperitoneum and lung are the most common sites for distant metastases.^7^ Renal metastases are very rare in all cancers, not just TGCT.^8^ The incidence of intraluminal thrombus at post chemotherapy RPLND is 5.8%, with approximately half containing active cancer or teratoma.^9^ This report presents a 35‐year‐old man with metastatic mixed testicular germ cell tumor to the para‐aortic lymph nodes and kidney who also developed renal vein thrombosis by the dominant component of teratoma, which is rarely reported in the literature review. Considering such a diagnosis is important for all surgeons treating patients with metastatic germ cell tumor to retroperitoneum or kidney, from the point of view of access to vascular surgery procedures, if needed.

## 2 CASE PRESENTATION

A 35‐year‐old man was admitted to the urology emergency department, in Imam Reza Hospital of Mashhad in Iran, with a chief complaint of an enlarged testicle on the left side. He had one child and no history of undescended testis in childhood. Initial physical examination revealed swelling of the left testis and the presence of a painless mass within the testis. Preoperative assessment of tumor markers revealed a significant increase in alpha‐fetoprotein (AFP: 685 ng/mL) (normal range, <40 ng/mL) and human chorionic gonadotropin (HCG: 2240 mIU/mL) (normal range, <2 mIU/mL). In addition, preoperative diagnostic computed tomography (CT) of the chest, abdomen, and pelvis showed no distant metastases. The patient then underwent radical inguinal orchiectomy in July 2018. Pathologic examination revealed the presence of a 13‐cm mass consisting of a yolk sac tumor (40%) and an immature teratoma component (60%). The neoplasm was confined to the testis and the tunica albuginea was free of tumor. No lymphatic invasion was noted. Five weeks after surgery, AFP and HCG had decreased to 132.6 ng/mL and 5.9 mIU/mL, respectively, and LDH levels were within the normal range (pathologic T1b, clinical N0 S1, AJCC prognostic stage: IS). The patient was referred to the oncology department. However, he refused further consultations.

**FIGURE 1:**
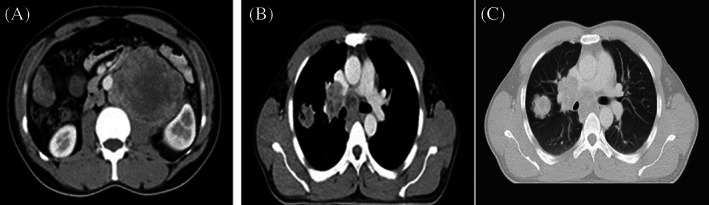
CT scan in September 2019 (at presentation in oncology department). (A) Axial plane of paraaortic lymph node at the level of L2–L3. (B) Axial plane of pathologic hilar and mediastinal lymph nodes at level of T6 (mediastinal window). (C) Axial plane of right pulmonary mass (lung window).

**FIGURE 2:**
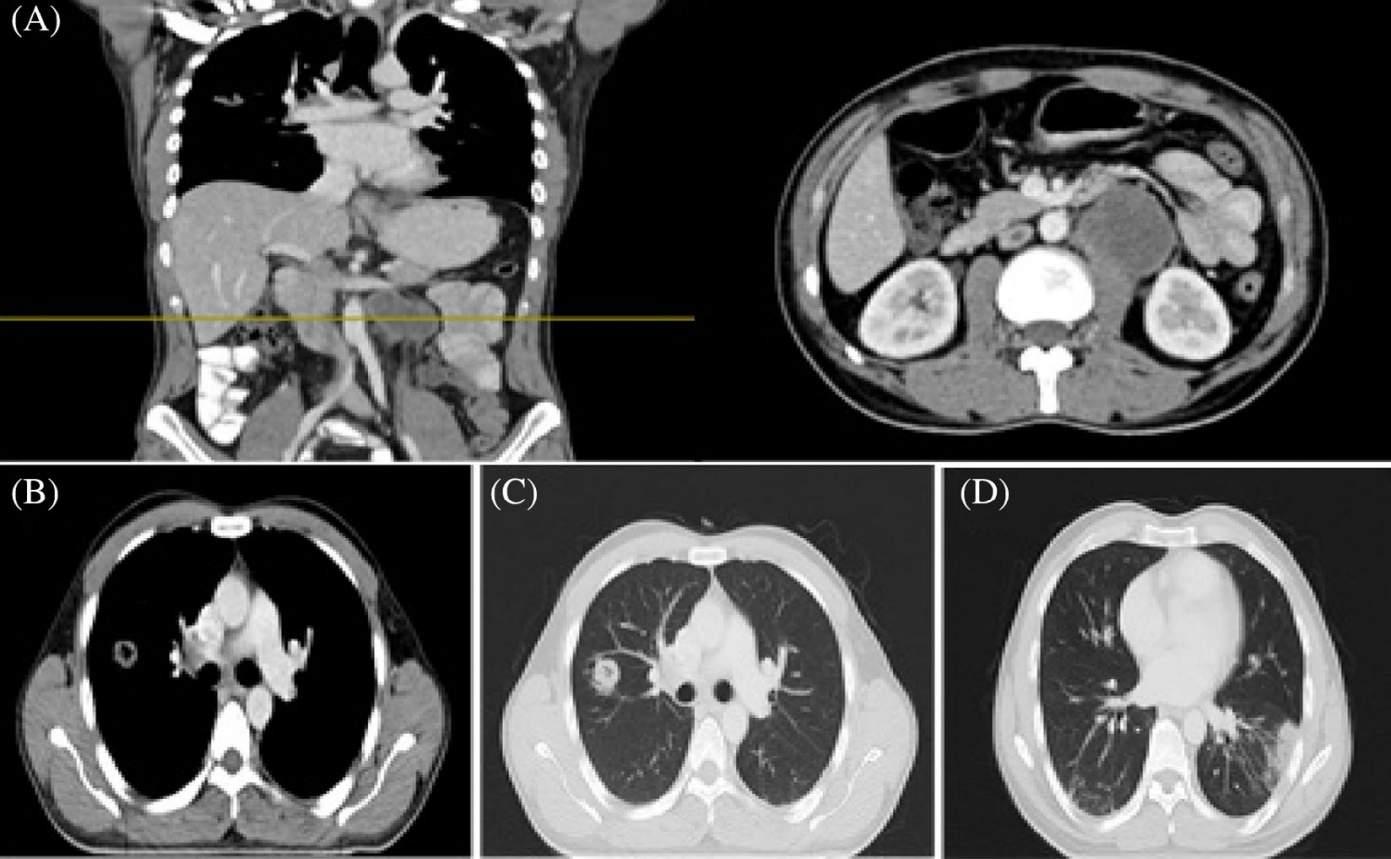
CT scan after 4 coarse BEP in December 2019. (A) Paraaortic lymph node at the level of L2–L3 (coronal and axial plane). (B) Axial plane of pathologic hilar and mediastinal lymph nodes at level of T6 (mediastinal window). (C) Axial plane of right pulmonary mass (lung window). (D) A new mass like lesion with ground glass opacity at posterior segment of left lower lobe.

In September 2019, the patient was admitted with persistent nonproductive cough, left flank pain, and low back pain. Serum levels of AFP and HCG were 543 ng/mL and 329 mIU/mL, respectively. In addition, a whole‐body CT scan revealed multiple mediastinal adenopathies, a mass with lobulated borders in the middle lobe of the right lung, and a 6‐cm left para‐aortic adenopathy that extended into the hilum of the left kidney along with a renal mass, which was suggestive of renal metastasis (Figure 1). The case was discussed by a multidisciplinary team, and chemotherapy with BEP every 21 days (bleomycin 30 U, days 1, 8, 15 + etoposide 100 mg/m^2^, days 1–5 + cisplatin 20 mg/m^2^, days 1–5) was initiated. After four cycles, serum levels of tumor markers normalized, renal and lung masses and para‐aortic lymphadenopathy decreased, but a new ground‐glass opacity appeared in the posterior segment of the left lower lobe, raising the differential diagnosis of recent pneumonia or a new metastatic lesion (December 2019) (Figure 2). With the diagnosis of teratoma‐predominant metastatic disease, the BEP regimen was replaced with VeIP (vinblastine sulfate 2 mg, days 1, 2 + ifosfamide 1200 mg/m^2^, days 1–5 + cisplatin 20 mg/m^2^, days 1–5) and surgical consultation was requested for resection of the metastases.

After 3 cycles, the size of the lung lesions decreased significantly, the mass lesion in the left lung disappeared, and para‐aortic lymphadenopathy and renal mass were stable. Tumor markers were also normal.

**FIGURE 3:**
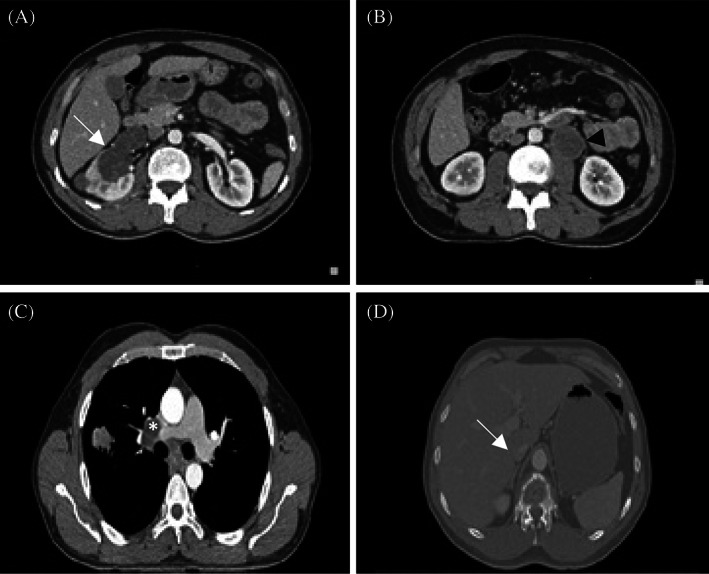
CT scan in December 2020 shows progression of all metastatic sites. (A) A 37 × 27 mm solid mass in medulla of the right kidney middle pole (arrow). (B) A 38 × 35 mm mass at the left paraaortic region (axial plane at the level of L2–L3) (arrow head). (C) Right lung mass, hilar, and mediastinal lymphadenopathies (axial plane at the level of T6). (D) Erosive lesion in T12 suggesting bone metastasis.

**FIGURE 4:**
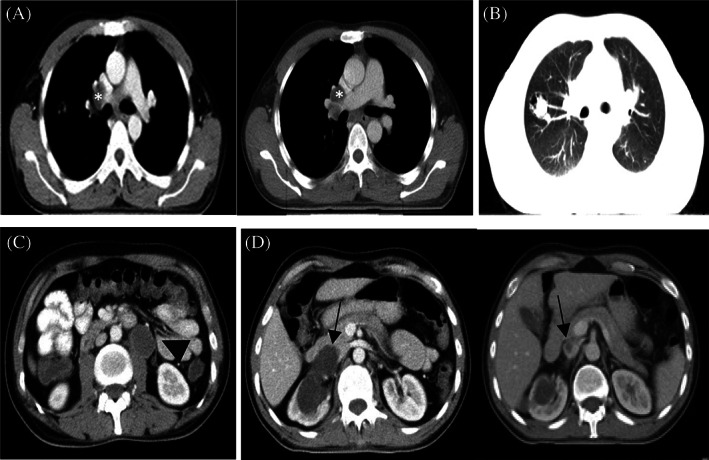
CT scan in March 2021. (A) Decreased size of lung mass, hilar, and mediastinal lymphadenopathies (asterisk). (B) 37 × 25 mm mass in upper lobe of right lung (parenchymal view). (C) A 34 × 27 mm paraaortic mass (arrow head). (D) A 77 × 33 mm cystic lesion with septa in middle pole of right kidney with extension to renal vein. The arrow shows IVC with a hypodense lesion in the center, which is actually along the tumoral thrombus of the renal vein.

However, for personal reasons, the patient did not adhere to surgical advice and did not visit the clinic for 12 months. In December 2020, he was hospitalized for worsening symptoms, and further examination revealed that all metastatic lesions had progressed significantly, in addition to a new lesion in the right renal medulla and an erosive lesion in the T12 vertebral body (Figure 3). Whole‐body bone scan also showed increased uptake of T12. Four cycles of chemotherapy with TIP (paclitaxel 250 mg/m^2^, day 1 + ifosfamide 1500 mg/m^2^, days 2–5 + cisplatin 25 mg/m^2^, days 2–5) were prescribed and completed in February 2021. After whole‐body CT scan (Figure 4) showed partial response of both lung mass and para‐aortic lymphadenopathy, the patient underwent radical nephrectomy and retroperitoneal lymphadenectomy (RN + RPLND) in June 2021. RN and RPLND were to be performed first, followed by thoracic metastatic resection and radiotherapy or surgery to remove the bone tumor if successful.

**FIGURE 5:**
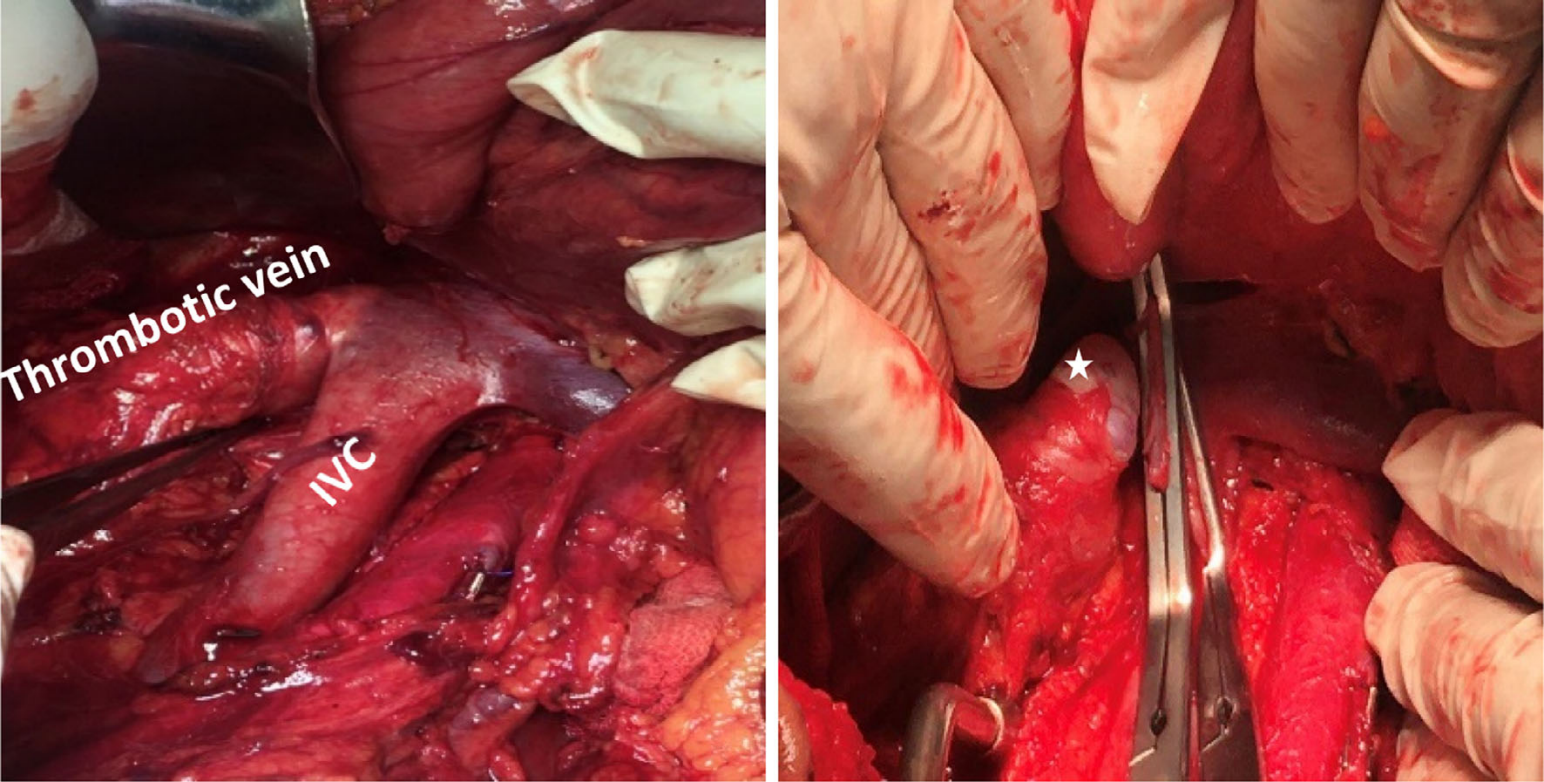
Macroscopic view of the renal vein tumoral thrombus in the operating room. The asterisk shows the cartilaginous gross appearance of tumoral thrombus, after cutting renal vein, with the dominant component of the teratoma.

**FIGURE 6:**
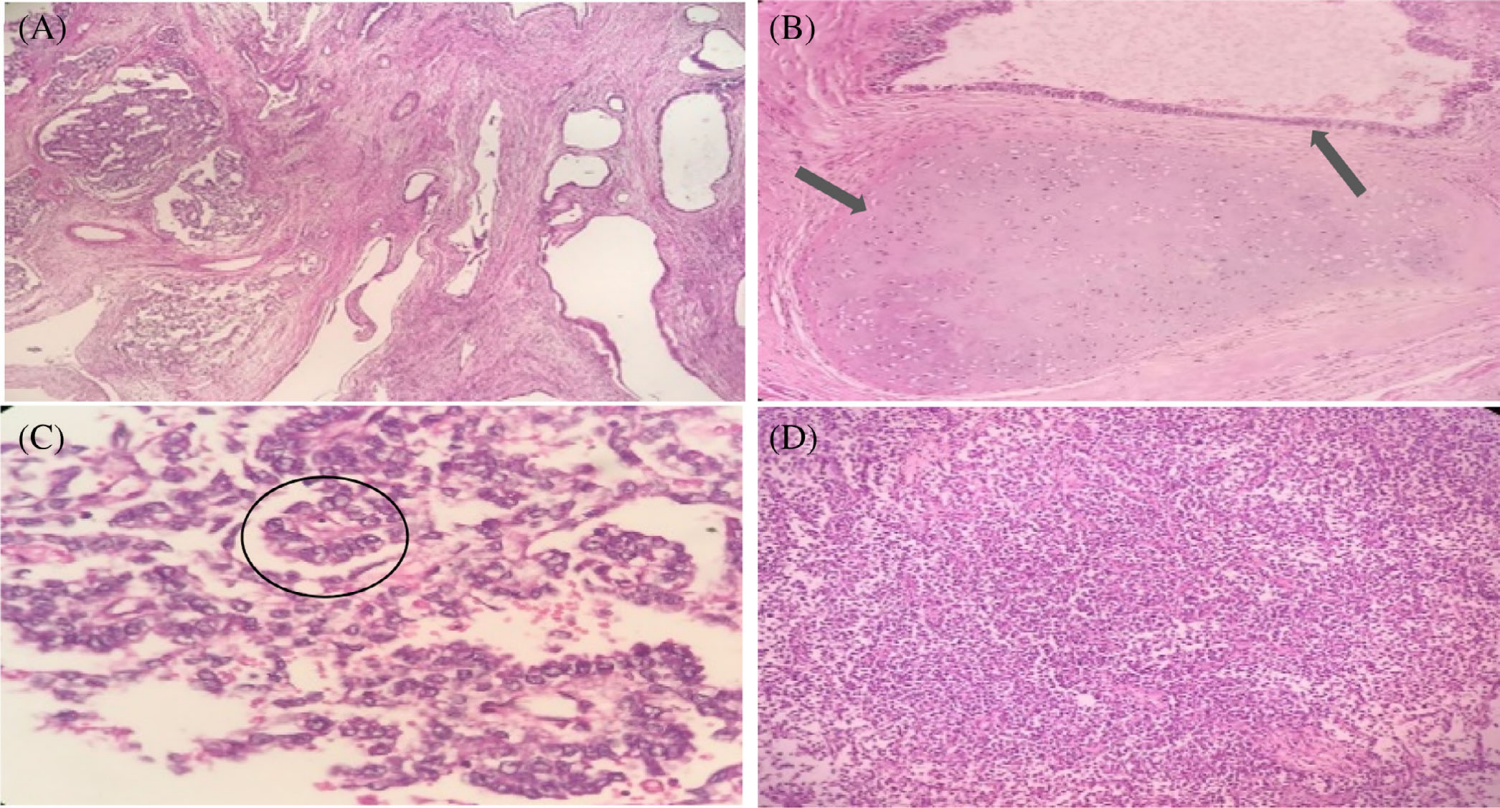
(A) Renal metastatic mixed germ cell tumor (H & E ×100). (B) Teratoma component (H & E ×100). (C) Yolk sac tumor component with perivascular arrangement of tumor cells (Schiller‐Duval body) (H & E ×400). (D) Seminoma; compact nests of large tumor cells (H & E ×100).

During the operation, renal vein thrombosis was detected by exploring the peritoneum. Therefore, radical nephrectomy was planned along with thrombectomy. First, the renal artery was cut, and then the renal vein was cut at the distal point of the tumor site. During the operation, the cartilaginous consistency of the renal vein thrombosis was noticed. Finally, the right kidney with a thrombosed renal vein was completely excised along with bilateral RPLND (Figure 5). Pathological findings revealed a solid cystic tumor mass measuring 4.5 × 3.5 × 2.5 cm located in the hilus of the left kidney and extending into the renal medulla, and microscopic examination revealed a metastatic germ cell tumor (teratoma: 70%, yolk sac tumor 25%, seminoma 5%) (Figure 6). In addition, resection of the left para‐aortic mass (size 8 × 4.5 × 3 cm) revealed necrotic tissue surrounded by a fibrohistiocytic reaction supporting necrotic lymph nodes. There was no viable tumor in the resected mass. After surgery, the patient's performance deteriorated and further surgery was not possible. He received T12 palliative radiotherapy (30 Gy/10 fractions) for pain control. Subsequently, three cycles of palliative chemotherapy with GemOx (gemcitabine 1000 mg/m^2^, days 1, 8 + oxaliplatin 130 mg/m^2^, day 1) were prescribed. Unfortunately, the response to treatment was not favorable, and the patient died after 3 months in January 2022.

## 3 DISCUSSION

In our patient tumoral involvement was seen in two sites that is rare manifestations of solid tumors. First, the kidney which is not a common site for distant metastases in all malignancies. Most studies of renal metastases are case reports.^10^, ^11^ Among them, metastatic germ cell tumor to the kidney is rare. The largest series was reported by Zhou et al.^12^ on 151 patients with renal metastases. The most common primary tumor was carcinoma and the most common primary tumor site was lung. Testicular tumors with distant renal involvement were not reported in this work. In another study by Chen et al.,^8^ 35 cases of renal metastasis were reported. Similar to the previous study, lung cancer was the most common primary tumor, and there were no reports of testicular cancer as the main cause of renal metastases. There are a few case reports of TGCT metastasizing to the kidney.^13^

Second, renal vein tumor thrombosis that according to autopsy reports, 11% of testicular tumors are associated with that, where as just 1% of them can be detected with imaging modalities.

There are several explanations for IVC thrombi such as tumor emboli, extension via the gonadal veins, external compression and occlusion of the vessel, direct invasion through the vessel wall, and lymphovenous shunting.^9^ Early detection of venous tumor thrombosis is important in point of prevention of complications such as pulmonary embolism, Budd‐Chiari syndrome, and cardiac complications.^14^

A rare feature of the present patient is the predominant teratomatous component of renal vein tumor thrombosis, for which there are no similar reports as far as we have searched. The embryonic component has been described as the major component of most tumor thrombosis.^14^, ^15^ Based on these data, despite their rarity, the possibility of renal metastasis in patients with TGCT should be considered either at baseline or during follow‐up. The surgeons must be aware of the risk factors that predict vascular involvement, and therefore, providing intraoperative access to vascular surgery procedures when needed. In situations where the neoplastic lesion is adherent to, or has intraluminal extension of the great vessels, vascular surgery procedures may be needed to be employed, in order to achieve complete tumor removal and better oncologic outcomes.^2^

Back pain and flank pain are the predominant preoperative symptoms in the present patient, possibly related to para‐aortic lymphadenopathy. Previous reports have shown that a substantial number of patients with renal metastases are asymptomatic regardless of their origin.^8^, ^12^, ^16^ Nevertheless, the most commonly reported complaints in symptomatic patients are back pain, flank pain, weight loss, and hematuria.^8^, ^12^ In this report, pathologic examination revealed a solid cystic tumor mass with intrarenal venous extension. Previous reports of renal metastases from TGCTs showed that cystic manifestation of this lesion is not uncommon in patients with metastatic renal germ cell tumors.^14^, ^15^ Regarding the intravascular extension of renal metastases, Raup et al.^13^ reported a similar finding. In their report, the patient had seminoma with right renal metastasis and tumor extension into the inferior vena cava. Choudhury et al.^17^ and Hadley et al.^18^ also reported other manifestations such as poorly defined nodular renal lesions within the inferior vena cava (IVC), thrombosis, or solid renal mass. While the main pathway for the occurrence of metastases is the infiltration of dedifferentiated cancer cells into the circulatory system and bloodstream,^16^ intravascular extension of tumor cells is not a common presentation due to loss of adhesion to the extracellular matrix, direct fluid shear stress, and immune system attack.^19^ However, certain protective mechanisms, such as tumor cell platelet microaggregates and inhibition of antitumor activity of natural killer cells, have been proposed to be responsible for intravascular survival of tumor cells.^20^

Regarding the treatment approach, the patient presented in our report was neglected because he did not seek adjuvant treatment after orchiectomy. However, given the stage IS and high APF at postoperative evaluation, three cycles of BEP out of four cycles EP were indicated.^20^ Active surveillance may be a viable option in these patients. However, patients should be aware of the possibility of recurrence and adhere to the principles of follow‐up CT scans.^21^ It is evident that more than 90% of recurrences occur in the first 2 years after orchiectomy, as it happened in our patient (disease‐free survival of 15 months).^22^ After disease progression, VeIp, TIP, and finally GemOx were successively administered, each of which produced some response. However, the prognosis for patients with metastatic TGCT is poor (<40% 5‐year survival),^23^ and the presented patient eventually had the overall survival of 41 months.

## 4 CONCLUSION

Renal metastases TGCT are very rare, but flank or low back pain in TGCT patients should be carefully investigated to rule out the possibility of renal metastasis. Determining the exact extent of malignant lesions, especially intrarenal vascular involvement, is crucial before treatment. The nature of treatment is palliative. However, surgery may be beneficial in patients with teratoma‐predominant TGCT, which is generally considered chemoresistant.

## AUTHOR CONTRIBUTIONS

**Hamidreza Ghorbani:** Conceptualization (lead); data curation (equal); investigation (lead); project administration (lead). **Ali Emadi Torghabeh:** Conceptualization (equal); data curation (equal); investigation (equal). **Mahdi Farzadnia:** Conceptualization (equal); data curation (lead); investigation (equal). **Alireza Golshan:** Conceptualization (equal); investigation (equal); project administration (equal). **Parisa Rabiei:** Conceptualization; data curation; supervision; writing – original draft; writing – review and editing.

## CONFLICT OF INTEREST STATEMENT

The authors have stated explicitly that there are no conflicts of interest in connection with this article.

## ETHICS STATEMENT

Written informed consent was obtained from the patient for his anonymized information to be published.

## Data Availability

Data of the reported case is available from the corresponding author upon request.

## References

[1] Park JS , Kim J , Elghiaty A , Ham WS . Recent global trends in testicular cancer incidence and mortality. Medicine. 2018;97(37):e12390. 30213007 10.1097/MD.0000000000012390 PMC6155960

[2] Evmorfopoulos K , Chasiotis G , Barbatis A , et al. Complete vascular replacement of the infrarenal inferior vena cava and abdominal aorta during post‐chemotherapy retroperitoneal lymph node dissection for a non‐seminomatous germ cell tumor. Curr Oncol. 2023;30(6):5448‐5455. 37366895 10.3390/curroncol30060412 PMC10296946

[3] Akyüz M , Topaktaş R , Ürkmez A , et al. Evaluation of germ‐cell neoplasia in situ entity in testicular tumors. Turk J Urol. 2019;45(6):418‐422. 29799399 10.5152/tud.2018.48855 PMC6788558

[4] Stang A , Rusner C , Trabert B , Oosterhuis JW , McGlynn KA , Heidinger O . Incidence of testicular tumor subtypes according to the updated WHO classification, North Rhine‐Westphalia, Germany, 2008‐2013. Andrology. 2019;7(4):402‐407. 30578617 10.1111/andr.12565 PMC8779128

[5] Williamson SR , Delahunt B , Magi‐Galluzzi C , et al. The World Health Organization 2016 classification of testicular germ cell tumours: a review and update from the International Society of Urological Pathology Testis Consultation Panel. Histopathology. 2017;70(3):335‐346. 27747907 10.1111/his.13102

[6] Howitt BE , Berney DM . Tumors of the testis: morphologic features and molecular alterations. Surg Pathol Clin. 2015;8(4):687‐716. 26612222 10.1016/j.path.2015.07.007

[7] Adra N , Althouse SK , Liu H , et al. Prognostic factors in patients with poor‐risk germ‐cell tumors: a retrospective analysis of the Indiana University experience from 1990 to 2014. Ann Oncol. 2016;27(5):875‐879. 26861605 10.1093/annonc/mdw045 PMC4843188

[8] Chen J , Qi N , Zhu S . Metastases to the kidney: an analysis of 35 cases and a review of literature. Front Oncol. 2020;10:632221. 33680955 10.3389/fonc.2020.632221 PMC7934622

[9] Johnston P , Beck SDW , Cheng L , et al. Incidence, histology and management of intraluminal thrombus at post‐chemotherapy retroperitoneal lymph node dissection. J Urol. 2013;190:874‐877. 23517745 10.1016/j.juro.2013.03.039

[10] Huo Z , Gao Y , Yu Z , Zuo W , Zhang Y . Metastasis of breast cancer to renal cancer: report of a rare case. Int J Clin Exp Pathol. 2015;8(11):15417‐15421. 26823905 PMC4713691

[11] Khan F , Mahmalji W , Sriprasad S , Madaan S . Prostate cancer with metastases to the kidney: a rare manifestation of a common disease. BMJ Case Rep. 2013;2013:bcr2012008388. 10.1136/bcr-2012-008388 PMC3762516 23907962

[12] Zhou C , Urbauer DL , Fellman BM , et al. Metastases to the kidney: a comprehensive analysis of 151 patients from a tertiary referral centre. BJU Int. 2016;117(5):775‐782. 26053895 10.1111/bju.13194 PMC4670601

[13] Raup VT , Johnson MH , Weese JR , Hagemann IS , Marshall SD , Brandes SB . Seminoma presenting as renal mass, inferior vena caval thrombus, and regressed testicular mass. Case Rep Urol. 2015;2015:835962. 25705542 10.1155/2015/835962 PMC4325215

[14] Kinebuchi Y , Ogawa T , Kato H , Igawa Y , Nishizawa O , Miyagawa SI . Testicular cancer with tumor thrombus extending to the inferior vena cava successfully removed using veno‐venous bypass: a case report. Int J Urol. 2007;14(5):458‐460. doi:10.1111/j.1442-2042.2007.01757 17511736

[15] Zarour CC , Zaki‐Metias KM , Gri J , et al. Testicular cancer with extensive gonadal and renal vein tumor thrombus. Clin Imaging. 2021;79:348‐352. doi:10.1016/j.clinimag.2021.07.022 34419852

[16] Feller L , Kramer B , Lemmer J . Pathobiology of cancer metastasis: a short account. Cancer Cell Int. 2012;12(1):24. 22676510 10.1186/1475-2867-12-24 PMC3407798

[17] Choudhury CR , Bhattacharya N , Bhutia TD , Mondal M . Mixed germ cell tumor in a teenager: a rare entity. Indian J Cancer. 2015;52(3):470‐472. 26905172 10.4103/0019-509X.176697

[18] Hadley DA , Cannon GH , Bishoff JT . A solitary seminoma renal metastasis presenting as an incidental renal mass. Urology. 2010;75(2):245‐246. 19660796 10.1016/j.urology.2009.05.068

[19] Strilic B , Offermanns S . Intravascular survival and extravasation of tumor cells. Cancer Cell. 2017;32(3):282‐293. 28898694 10.1016/j.ccell.2017.07.001

[20] Gilligan T , Lin DW , Aggarwal R , et al. Testicular cancer, version 2.2020, NCCN clinical practice guidelines in oncology. J Natl Compr Canc Netw. 2019;17(12):1529‐1554. 31805523 10.6004/jnccn.2019.0058

[21] Albers P , Albrecht W , Algaba F , et al. Guidelines on testicular cancer: 2015 update. Eur Urol. 2015;68(6):1054‐1068. 26297604 10.1016/j.eururo.2015.07.044

[22] Winter C , Hiester A . Treatment of clinical stage I non‐seminoma. Asian J Urol. 2021;8(2):161‐169. 33996471 10.1016/j.ajur.2021.03.001 PMC8099697

[23] Kollmannsberger C , Nichols C , Meisner C , Mayer F , Kanz L , Bokemeyer C . Identification of prognostic subgroups among patients with metastatic ‘IGCCCG poor‐prognosis’ germ‐cell cancer: an explorative analysis using cart modeling. Ann Oncol. 2000;11(9):1115‐1120. 11061604 10.1023/a:1008333229936

